# Hypofractionated stereotactic radiotherapy (HFSRT) versus single fraction stereotactic radiosurgery (SRS) to the resection cavity of brain metastases after surgical resection (SATURNUS): study protocol for a randomized phase III trial

**DOI:** 10.1186/s12885-023-11202-9

**Published:** 2023-07-29

**Authors:** Maria Waltenberger, Denise Bernhardt, Christian Diehl, Jens Gempt, Bernhard Meyer, Christoph Straube, Benedikt Wiestler, Jan J. Wilkens, Claus Zimmer, Stephanie E. Combs

**Affiliations:** 1 Department of Radiation Oncology, Klinikum rechts der Isar, Technical University of Munich (TUM), Ismaninger Straße 22, 81675 Munich, Germany; 2 German Cancer Consortium (DKTK), Partner Site Munich, Munich, Germany; 3 Department of Neurosurgery, Klinikum rechts der Isar, Technical University of Munich (TUM), Ismaninger Straße 22, 81675 Munich, Germany; 4 RADIO-LOG Hof, Eppenreuther Straße 9, 95032 Hof, Germany; 5 Institute of Neuroradiology, Klinikum rechts der Isar, Technical University of Munich (TUM), Ismaninger Straße 22, 81675 Munich, Germany; 6 Institute of Radiation Medicine (IRM), Helmholtz Zentrum, Ingolstädter Landstraße 1, 85764 Neuherberg, Germany

**Keywords:** Brain metastases, Resection cavity, Local control, Stereotactic radiotherapy, Radiosurgery, Clinical trial, Randomized trial

## Abstract

**Background:**

The brain is a common site for cancer metastases. In case of large and/or symptomatic brain metastases, neurosurgical resection is performed. Adjuvant radiotherapy is a standard procedure to minimize the risk of local recurrence and is increasingly performed as local stereotactic radiotherapy to the resection cavity. Both hypofractionated stereotactic radiotherapy (HFSRT) and single fraction stereotactic radiosurgery (SRS) can be applied in this case. Although adjuvant stereotactic radiotherapy to the resection cavity is widely used in clinical routine and recommended in international guidelines, the optimal fractionation scheme still remains unclear. The SATURNUS trial prospectively compares adjuvant HFSRT with SRS and seeks to detect the superiority of HFSRT over SRS in terms of local tumor control.

**Methods:**

In this single center two-armed randomized phase III trial, adjuvant radiotherapy to the resection cavity of brain metastases with HFSRT (6 – 7 × 5 Gy prescribed to the surrounding isodose) is compared to SRS (1 × 12–20 Gy prescribed to the surrounding isodose). Patients are randomized 1:1 into the two different treatment arms. The primary endpoint of the trial is local control at the resected site at 12 months. The trial is based on the hypothesis that HFSRT is superior to SRS in terms of local tumor control.

**Discussion:**

Although adjuvant stereotactic radiotherapy after resection of brain metastases is considered standard of care treatment, there is a need for further prospective research to determine the optimal fractionation scheme. To the best of our knowledge, the SATURNUS study is the only randomized phase III study comparing different regimes of postoperative stereotactic radiotherapy to the resection cavity adequately powered to detect the superiority of HFSRT regarding local control.

**Trial registration:**

The study was retrospectively registered with ClinicalTrials.gov, number NCT05160818, on December 16, 2021. The trial registry record is available on https://clinicaltrials.gov/study/NCT05160818. The presented protocol refers to version V1.3 from March 21, 2021.

## Background

Depending on the underlying tumor, up to 30 per cent of all cancer patients develop brain metastases in the course of their disease [1]. Without any treatment, the survival after diagnosis of symptomatic brain metastases is only about a few weeks to months [2, 3]. Since the risk of local recurrence is very high with resection alone [4], postoperative radiotherapy is recommended. However, there is controversy about the best treatment regimen. Many centers prefer adjuvant radiotherapy of the resection as local treatment over whole brain radiotherapy (WBRT) as a standard procedure, due to the superiority in terms of neurocognitive side effects and comparable survival [5].

Adjuvant local irradiation is well established in the context of limited brain metastases [5–8], yet data regarding extensive brain disease (> 4 metastases) is emerging [9–11]. Its use is more frequently recommended in international guidelines such as the NCCN National Comprehensive Cancer Network® Guideline Central Nervous System Cancers V1.2022. Despite intensive research, the optimal dose prescription of adjuvant radiotherapy to the resection cavity remains unclear. The resection cavity can be treated in few fractions (hypofractionated stereotactic radiotherapy, HFSRT), or with single dose radiosurgery (SRS). Both regimen are used in clinical routine, and every center may have their preference, or apply both, depending on cavity volume, location, or other patient-related factors. When determining the dose, a balance must be struck between tumor control and the risk of radionecrosis in particular. Data available on radiotherapy of the resection cavity is mostly retrospective in nature. There exist only two recent prospective adequately powered trials on stereotactic irradiation of the resection cavity: Both the trial by Mahajan et al. [5] comparing adjuvant local irradiation to observation, and the trial by Brown et al. [6] that compared adjuvant local treatment to WBRT, applied SRS. Within the prospective trial by Mahajan et al. 63 patients randomized in the treatment arm were irradiated with 1 × 12–16 Gy prescribed on the 50% isodose line, applying a 1 mm safety margin. Irradiation was carried out with a Leksell Gamma Knife, using a stereotactic head frame for patient positioning. Patients with a  maximum of three resected metastases with a cavity size of ≤ 4 cm were included in the trial. Reporting no event of radiation necrosis, local control (LC) of the irradiated resection cavity at 12 months was 72%, which was significantly superior to observation alone. Brown et al. included patients with one resected brain metastasis and a maximum of four brain metastases in their prospective trail. The 98 patients randomized into the SRS arm received 1 × 15–20 Gy with a safety margin of 2 mm. The irradiation dose was prescribed to the highest isodose line encompassing the planning target volume (PTV). Maximum cavity diameter was 5 cm. At 12 months after SRS, LC and radionecrosis rate were 61% and 4%, respectively. The trial was able to show that WBRT significantly yields more cognitive deterioration without improving overall survival compared to SRS. For SRS, safety margins are generally smaller than for fractionated regimens, which may likely be responsible for the relatively higher local failure rate in the single-dose trials. For reproducibility, an approach recommending standardized contouring of the surgical cavity for stereotactic radiosurgery has been published recently [12].

However, the published treatment regimens of our own institution by Specht et al. [13], applying 35 Gy in 7 fractions to the 95–100% isodose line, were demonstrated to be not only feasible and safe, but also superior in terms of LC compared to the results of the beforementioned prospective trials, reporting a 1-year LC of 88%. A favorable LC rate after HFSRT was also shown in a multicenter analysis by Combs et al. [14], reporting a crude LC rate of 80.5% after adjuvant radiotherapy to the resection cavity with a median dose of 35 Gy (range 20–42 Gy) in 7 fractions (range 4–14). The hypothesis of superiority of HFSRT is further supported by the results of a recently published international multicenter analysis by Eitz et al. [15] demonstrating a 1-year LC rate of 84% after irradiation with a median total dose of 30 Gy (range 18–35 Gy) and a dose per fraction of 6 Gy (range 5–10.7 Gy). So far, it exists only one broad meta-analysis that revealed significantly improved LC rates after HFSRT compared to SRS [16] (pooled rate of LC at 12 months of 87.3% vs 80.0%, *p* = 0.021). The observed superiority of HFSRT in terms of LC may be due to fractionation, differences in target volumes i.e., safety margins, or other. However, which treatment is superior can only be clarified within a prospective randomized trial. We therefore designed the SATURNUS study comparing both treatment regimens in a standardized and prospective way.

## Methods/Design

### Aim of the trial

Based on our own institutional data [13] and a recently published meta-analysis [16], we hypothesize that LC after HFSRT is superior compared to LC after SRS. With this study, we seek to prove the superiority of HFSRT in terms of LC. To the best of our knowledge, the SATURNUS study is the only randomized phase III study comparing different techniques of postoperative stereotactic radiotherapy after resection of brain metastases adequately powered to detect a superiority of HFSRT regarding LC.

### Trial registration

The study was retrospectively registered with ClinicalTrials.gov, number NCT05160818, on December 16, 2021. The trial registry record is available on https://clinicaltrials.gov/study/NCT05160818. The presented protocol refers to version V1.3 from March 21, 2021. Important protocol modifications, if applicable, will be published in the study’s entry on ClinicalTrials.gov.

### Trial population

Patients with up to three resected brain metastases and possibly further brain metastases for which there is no indication for surgery.

### Primary endpoint

- Rate of LC at the resected site(s) at 12 months

### Secondary endpoints

- Rate of LC at all treated site(s) at 12 months
- Rate of locoregional control (LRC) at 12 months
- Overall survival (OS)
- Salvage-free survival
- Number and kind of intracranial salvage treatments
- Rate of pseudoprogression
- Irradiation-related toxicity according to CTCAE v4.03, especially rate of radionecrosis
- Quality of life according to EORTC QLQ-C30 and EORTC QLQ-B20
- Time to loss of independence defined as decrease in Barthel index by > 20 points

### Inclusion criteria

- Histologically confirmed solid tumor disease
- One to three resected brain metastases
- Consent to perform adjuvant irradiation by an interdisciplinary tumor board
- Completed wound healing
- Resection within the last six weeks at the time of study inclusion
- Diameter of the resection cavity ≤ 4 cm (on Planning MRI)
- Age ≥ 18 years
- Karnofsky Performance Score (KPS) ≥ 60%
- Adequate contraceptive measures for fertile women / men
- Written informed consent (must be available before enrolment in the trial)

### Exclusion criteria

- Contraindication for repetitive contrast enhanced MRI
- Leptomeningeal disease
- Small cell histology, hematological malignancies and / or germ cell malignancies
- Previous irradiation of the brain
- Pregnant and lactating women
- Inability to understand the character and consequences of the study
- Withdrawal of consent

### Trial design

**Fig. 1:**
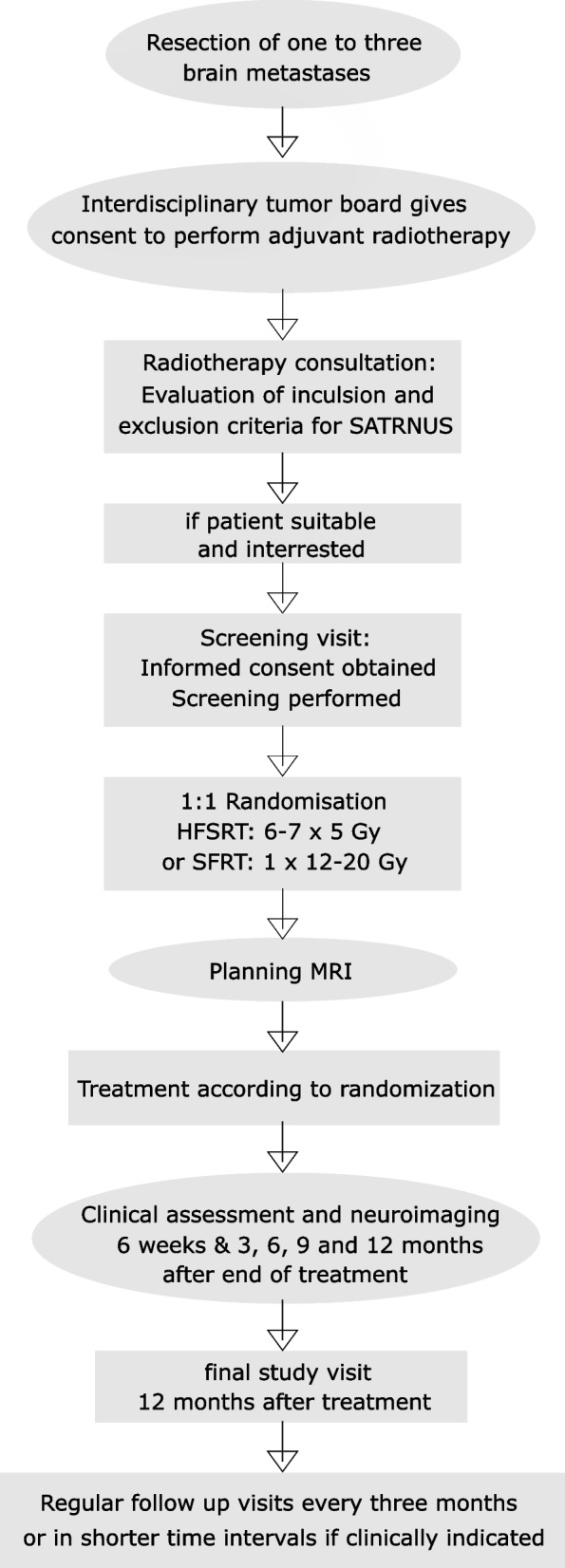
Flow chart of the SATURNUS/NCT05160818 trial

The trial is designed as a prospective, randomized, controlled, monocentric phase III trial. It is conducted at the academic hospital Klinikum rechts der Isar of the Technical University of Munich, Germany. A flow chart of the study is provided in Fig. 1. Patients fulfilling the above criteria are considered for study participation and participants are randomly 1:1 allocated to either adjuvant HFSRT (6 – 7 × 5 Gy i.e., 30 Gy/6 fractions or 35 Gy/7fractions; Arm A) or SRS (1 × 12–20 Gy; Arm B). In case a higher dose is needed for adequate tumor control (at the discretion of the treating physician), the maximum allowed dose depends on the cavity diameter and does equivalate the findings of the RTOG 90–05 trial (24 Gy, 18 Gy, and 15 Gy for tumors ≤ 20 mm, 21–30 mm, and 31–40 mm in maximum diameter) [17]. Unresected brain metastases, if present, are treated with SRS (1 × 14 – 22 Gy, prescribed to the surrounding isodose). The affiliation to the treatment arm will not be blinded to anyone except the study neuroradiologist.

### Randomization

Patients are randomized 1:1 into the two treatment arms using a randomization list generated with Randomizer, a randomization service for clinical trials, available free of charge at https://randomizer.at. Permuted block randomization with a block size of eight is applied. Sealed envelopes are used and opened by the responsible study nurse or study physician. Assignment to a therapy device is not subject to randomization but is analogous to clinical practice.

### Treatment devices, patient positioning and image guidance

Irradiation is carried out with a Leksell Gamma Knife (Elekta, Stockholm, Sweden) or a Linear Accelerator dedicated for high-precision radiotherapy. For SRS at the Gamma Knife, two methods of patient positioning are available: stereotactic mask or stereotactic frame. In an informed consent procedure, the patient chooses the fixation method himself/herself. For HFSRT at the Gamma Knife, only mask fixation is offered due to the reproducibility on the different treatment days. For image guidance, a stereotactic ConeBeam-CT is used. Besides, during irradiation, an infrared tracking system (High Definition Motion Management, HDMM, Elekta, Stockholm, Sweden) is used. For irradiation at the Linear Accelerator, patient positioning will be carried out using a thermoplastic mask system as otherwise technically not feasible. Precise dose application is ensured with the ExacTrac system (X-ray-based positioning system, Brainlab, Germany).

### Dose prescription

- Arm A HFSRT: 6 – 7 × 5 Gy prescribed to the surrounding isodose
- Rationale: Dose regimen is analogous to the own institutional data by Specht et al. [13] yielding excellent local control rates after adjuvant stereotactic irradiation with 7 x 5 Gy on which the sample size calculation is based (see below). It has been shown that 6 x 5 Gy and 7 x 5 Gy lead to comparable tumor control rates in adjuvant stereotactic irradiation of the resection cavity [14], and therefore dose de-escalation to 6 x 5 Gy may be allowed, if desired/needed for protection of structures at risk
- Arm B SRS: 1 × 12-20 Gy prescribed to the surrounding isodose, depending on cavity size and proximity to structures at risk.
- Rationale: Dose regimen for SRS are analogous to the dose regimen applied in the phase III trials by Mahajan et al. [5] and Brown et al. [6], on which the sample size calculation is based (see below). Mahajan et al. applied SRS with 1 x 12-16 Gy, whereas Brown et al. delivered postoperative SRS with 1 x 15-20 Gy.
- In case a higher dose is needed for adequate tumor control (at the discretion of the treating physician), the maximum allowed dose depends on the cavity diameter and does equivalate the findings of the RTOG 90-05 trial (24 Gy, 18 Gy, and 15 Gy for tumors ≤ 20 mm, 21-30 mm, and 31-40 mm in maximum diameter, respectively) [18]
- Unresected brain metastases, if present, are treated with SRS: 1 × 14 – 22 Gy prescribed to the surrounding isodose. In individual cases (e.g., metastasis near the brain stem), a fractionated approach with, for example, 6 × 5 Gy may be necessary.

### Dose constraints

Structures at risk such as the brain stem, optic nerves, chiasm, and spinal cord will be contoured. Dose constraints of normal tissue will be respected according to the Quantitative Analyses of Normal Tissue Effects in the Clinic (QUANTEC) recommendation [19]. Radiotolerance of adjoining structures at risk can be limiting to dose prescription and can in certain cases require a slight reduction of the abovementioned dose regimen. For healthy tissue special consideration will be given to the healthy brain, which is being defined as structure at risk (healthy brain = whole brain – sum of all PTVs).

### Target volume definition

Treatment planning MRI is conducted within seven days prior to the start of irradiation for stereotactic mask fixation and on the day of treatment for stereotactic ring fixation. To ensure a uniform target volume definition approach and in accordance with a current consensus paper by Soliman et al. [12] the resection cavity (RC) including all contrast-enhancing lesions (T1w image after gadolinium contrast) and the surgical tract will be defined as resection cavity volume (RCV). In case of partial resection or local recurrence between resection and treatment planning MRI, all tumor remnants are included in the RCV.

For HFSRT, RCV will be expanded by up to 5 mm (depending on site, up to the treating physician’s discretion) to account for microscopic invasion to generate the CTV. For SRS, RCV will be expanded by 1 mm for CTV generation. Edema is generally not included into the CTV.

For PTV formation, a 1 mm margin will be used for HFSRT and SRS with mask positioning to account for inter-fractional cavity dynamics [20] as well as potential positioning uncertainties. In SRS with stereotactic frame positioning, PTV will equal CTV.

### Follow-up

FU visits consist of regular clinical visits and MRI scans. They will be performed at 6 weeks and three, six, nine and twelve months after the end of treatment, and additionally upon request or if new symptoms develop. Clinical follow-up includes the assessment of the following parameters/data:

- oncologic and non-oncologic medical history: staging examinations, chronic and acute diseases, interventions, and symptoms
- Karnofsky performance score, BMI
- disease related physical examination
- disease related symptoms
- oncologic and non-oncologic medication (the parameter “corticosteroid use” is also collected for the final evaluation of the therapy response done by the radiotherapist)
- questionnaires: EORTC QLQ-C30, EORTC QLQ-B20 and Barthel-Index
- schematic assessment of the MRI scan findings (RAF): measurements of every treated lesion, measurements of possible new lesions, final evaluation of the therapy response

### Assessment of local control

FU images are interpreted by the study neuroradiologist based on the response assessment criteria as proposed by the Response Assessment in Neuro-Oncology Brain Metastases (RANO-BM) group [21], but slightly modified to accommodate the objective of this trial. Radiographical response will be measured unidimensional and will be recorded for every resected lesion. In case of tumor recurrence, the first MRI scan suspicious of tumor progression is considered as the timepoint of recurrence. The diameter of the resection cavity and possible cystic components are not measured for response assessment.

LC will be evaluated separately for every resected lesion at each follow up MRI. For the separate assessment of LC of each specific resected lesion, use of corticosteroids and clinical condition are not considered, as this could lead to a confounding of the results.

In accordance with the proposal of the RANO-BM group, resected lesions are allocated to either measurable or non-measurable disease based on their appearance on the treatment planning MRI.

Lesions are considered measurable if they fulfil all the following criteria:

- nodular contrast-enhancement (as component of the RC)
- visible on two or more axial slices
- measurable in at least one dimension with a minimum size of 5 mm (LD)

Any other lesions are classified as non-measurable.

For response assessment, the following criteria are applied for:

- Measurable lesions ≥ 5 mm and < 10 mm:
  - ◦ CR = no contrast enhancement visible
  - ◦ PR = minimum 3 mm decrease in longest diameter compared to baseline (treatment planning MRI)
  - ◦ SD =  < 3 mm increase or decrease in longest diameter
  - ◦ PD = minimum 3 mm increase in longest diameter compared to nadir
- Measurable lesions ≥ 10 mm:
  - ◦ CR = no contrast enhancement visible
  - ◦ PR = minimum 30% decrease in longest diameter compared to baseline (treatment planning MRI)
  - ◦ SD =  < 30% decrease or < 20% increase in longest diameter
  - ◦ PD = minimum 20% increase in longest diameter compared to nadir
- Non-measurable lesions:
  - ◦ Exact measurement of non-measurable lesions is not required
  - ◦ Non-measurable lesions should be classified as absent, present or unequivocal progression

The final response assessment is done by the study radiotherapist according to the following criteria:

- CR can only be stated if there is no corticosteroid use related to brain metastases and if the clinical status related to brain metastases is stable or improved
- PR can only be stated if the corticosteroid use related to brain metastases is stable or reduced and if the clinical status related to brain metastases is stable of improved
- If SD or PD of pre-existing lesions has been stated by the study neuroradiologist, clinical information cannot induce a change in the final response assessment done by the radiotherapist

In case of suspected radiation necrosis, diagnostic strategies include repeat MRT exam within 6 weeks as well as advanced imaging techniques (perfusion MRI, 18F-FET-PET scan, APTw imaging) and ultimately surgical pathology via biopsy or resection. When radiation necrosis is suspected in a follow up imaging, interdisciplinary case discussion at a neurooncologic tumor board is mandatory.

### Statistical considerations and sample size calculation

The underlying null hypothesis of this study is that there is no difference in terms of LC between HFSRT and SRS. The aim of the study is to provide evidence that allows to refute the null hypothesis with a certainty of at least 95% (95% CI), so that the alternative hypothesis can be considered valid. The alternative hypothesis is that HFSRT is superior to SRS in terms of LC. The underlying data for case number calculation are the prospective trials by Mahajan et al. [5] and Brown et al. [6] for SRS (pooled mean LC rate of 66% at 12 months) and our own institutional retrospective data for HFSRT [13] (mean LC rate of 88% at 12 months). The sample size calculation was performed with a two group Chi-squared test of equal proportions (odds ratio = 1). The test significance level (α) was set to 0.05. Allocating an equal number of patients to both treatment arms, 114 patients are needed to detect a superiority of HFSRT with a power of at least 80%. We estimated a dropout rate of 10%, resulting in a total number of 126 patients, and a monthly accrual of three patients. Patients will be randomized 1:1 in the two different treatment arms. Logistic regression analysis will be used to perform predefined subgroup analyses with respect to the primary endpoint.

### Analysis plan

The primary analysis will be based on the full analysis set which is according to the intention-to-treat principle. The Intention to treat Population includes all patients that were randomized into one of the treatment arms, disregarding the actual treatment, possible non-compliance or withdrawal that may happen after randomization. We have chosen this approach as the method of primary analysis because it better reflects actual clinical processes, knowing that the analysis results concerning the treatment effect are more likely to be conservative.

Interim Analysis: An interim analysis is planned after the first 40 patients have been treated and followed up for 12 months. It mainly serves as a safety analysis. SAEs and AEs in both treatment arms will be compared. A statistically significant difference in SAEs related and probably related to the study therapy between the treatment arms will lead to a premature closure of the study.

### Subgroup analyses

Apart from fractionation schemes, there exist other parameters that might influence LC after RC irradiation. To evaluate possible further influencing factors, the PubMed database (https://pubmed.ncbi.nlm.nih.gov) was searched for the combined criteria "brain metastases", "resection cavity irradiation" and "local control". The literature search yielded 137 articles. After abstract review, 13 articles remained that appeared suitable to deliver relevant information, all retrospective in nature. There is no parameter that has consistently been demonstrated as a significant influencing factor on LC. However, those factors that showed a significant influence of LC in at least one dataset have been included as stratification factors for subgroup analyses. Subgroup analyses will be carried out using logistic regression analysis. The influencing factors identified are listed below, together with the supporting literature:

1. Histology: Radioresistant vs. radiosensitive primary.
  Although a radioresistant histology such as melanoma, colorectal carcinoma or sarcoma usually does not necessarily appear to have a significant impact on LC [22], Soliman et al. [23] detected a significant association between radioresistant primary and reduced LC.
2. Preoperative tumor volume: metastases (also) resected for size vs. metastases resected for clinical complaints only.
  Preoperative tumor size has also been described as a significant factor influencing local recurrence [24, 25]. A uniform cut-off value was not observed, therefore the categories "resected for size" (according to our in-house standard operating procedures approx. 3 cm in diameter) and "resected for clinical complaints" were defined for this subgroup analysis.
3. Extent of resection (assessed on the planning MRI): residual tumor vs. complete resection.
  As shown by El Shafie et al. [26], incomplete resection may significantly lower LC rates.
4. Size of RC (assessed on the planning MRI): Size > median vs. ≤ median.
  The size of the resection cavity or the PTV volume has been demonstrated to significantly influence LC in several data sets [7, 15, 24–28], with bigger RC yielding poorer LC rates. Although absolute cut-off values differ, they are close to the median resection cavity volume / PTV volume in the respective data sets. In the SATURNUS trial, the size of the PTV is also dependent on the allocation to the treatment arm and positioning method. Therefore, the extent of the RC is the more appropriate stratification factor for this subgroup analysis.
5. Number of brain metastases: > 1 vs. 1
  As shown by Eitz et al. [15] > 1 metastasis present can have a significantly negative impact on LC.
6. Extracranial disease status: uncontrolled primary tumor vs. controlled primary tumor at time of Screening Visit.
  In the multi-institutional analysis by Eitz et al. [15], an uncontrolled primary tumor yielded poorer LC rates.
7. Dose: Margin dose < 48 Gy BED10 vs. >48 Gy BED10.

There are a couple of analyses [24, 28, 29] showing a significant impact of delivered dose on LC. As 48 Gy BED10 has been demonstrated as cut-off value in two independent data sets [28, 29], this threshold is also used in the subgroup analysis.

Regarding the primary endpoint (LC of resected metastases) as well as LC of non-resected metastases, it will be analyzed exploratory whether an association between LC rate and prescription IDL as well as Dmax, Dmean and Dmin of the PTV can be observed. For the secondary endpoints LRC, OS and development of radionecrosis and pseudoprogression, possible influencing factors will be analyzed exploratively.

### Recruitment and trial duration

Recruitment of patients started in May 2021. The first patient was enrolled on 05/06/2021. The trial is currently recruiting. After completion of the recruitment phase, a minimum FU period of 12 months is planned. Estimating a recruitment rate of three patients per month, the projected time to complete the study will be 4.5 years.

### Subject discontinuation/withdrawal and data handling

A subject may voluntarily discontinue participation in this study at any time at his/her own request. In this case a justification is not mandatory. If further study participation is refused, data already obtained will either be deleted or evaluated (and then included in the study evaluation) at the request of the subject. The study subject has the right to have his/ her data deleted in accordance with Article 17 of the DSGVO (German General Data Protection Regulation). However, in accordance with Article 7, paragraph 3 of the DSGVO, the lawfulness of data processing that has already been carried out is not affected by such a revocation.

Once recruitment is complete and all patients have been followed up for at least 12 months, the database will be declared as closed (data lock). The statistical analysis of the study will start after data lock. This does not include the interim analysis.

According to §13 Section 10 of the Good Clinical Practice Regulation, all trial documents will be archived for at least 10 years after the termination of the study (last patient last visit). According to §85 Section 2 of the German Radiation Protection Act (StrSchG), records of radiographs, digital image data and other examination data will be kept for 30 years after the last treatment (last day of study treatment). The Study Center (Department of Radiation Oncology, Klinikum rechts der Isar, Technical University of Munich) is responsible for data archiving and for providing adequate data storage facilities.

### Confidentiality, protocol amendments and trial publication

The names and personal information of study participants will be held in strict confidence. All study records (case report forms, safety reports, correspondence, etc.) will only identify the subject by initials and the assigned study identification number. A confidential subject identification list (Master List) will be maintained during the course of the study. Access to confidential information (i.e., source documents and patient records) is only permitted for direct subject management and for those involved in monitoring the conduct of the study. The subject’s name will not be used in any public report of the study.

Any modification to the trial protocol must be approved by the SC and PI and can only be declared admissible after positive approval by the IRB. Protocol amendments will be communicated to all investigators and trial registries by the SC. The current version of the protocol is V1.3 from March 21, 2021. All information concerning the trial is confidential before publication. Any communication or publication of trial results will be led by the SC and the PI. Trial results will be published independent of the results.

## Discussion

Stereotactic irradiation to the resection cavity is a well-established and far recommended treatment strategy after resection of brain metastases [30]. It’s superiority over observation alone in term of LC has been demonstrated prospectively (72% vs. 43%, *p* = 0,015) [5]. Regarding cognitive side effects, it is better tolerated than WBRT without compromising overall survival (cognitive deterioration free survival 3.7 vs 3 months, *p* < 0.0001) [6]. Despite being considered standard of care treatment, there is a need for further prospective data on adjuvant stereotactic irradiation to the resection cavity, especially to be able to optimize the treatment concepts in detail. Both SRS and HFSRT are possible treatment options and reported LC rates and toxicities vary substantially between different patient cohorts and institutions. We found a striking difference in prospectively reported LC rates after SRS as opposed to our own institutional data on HFSRT [13, 27, 31]. Being further supported by a recently published meta-analysis by Akanda et al. [16] reporting a significantly higher LC rate at 12 months after HFSRT (87,3% vs 80%, *p* = 0.021), we designed the presented SATURNUS study to prospectively demonstrate the superior LC rates after HFSRT. Since the treated brain metastases themselves rarely lead to death, LC, but also therapy side effects after stereotactic irradiation, especially radionecrosis, are of particular relevance. As outlined above, there might be several factors that contribute to the efficacy and toxicity of the treatment. One of those might likely be the delivered radiation dose.

The SATURNUS study is a prospective, randomized, controlled phase III trial at this point of time planned as a monocentric study that seeks to accrue 126 patients with up to three resected brain metastases to receive adjuvant stereotactic irradiation. It aims to determine the optimal fractionation scheme (HSFRT vs. SFRT) regarding local tumor control. Side effects, among others, are evaluated as secondary endpoints. The SATURNUS study is based on the hypothesis of the superiority of HFSRT to SRS regarding LC, based on our own institutional data [13] on HFSRT in comparison with the available prospective phase III data on SRS [5, 6]. To also consider other possible influencing factors on LC, the SATURNUS study included a broad subgroup analysis of factors identified in a literature search. One of these factors, among many others, is radiation dose. We provided the rationale for the dose schemes allowed within the study yet acknowledge that the dose prescriptions allowed within the study are broad, especially for SRS. Some retrospective analyses, for both SRS and HFSRT, observe no significant effect of dose on local control [14, 15, 23, 26, 32–34]. On the other hand, in the prospective phase III trail by Mahajan et al. [5], LC was observed to be significantly lower with larger cavities. As per protocol, larger cavities receive less dose. However, it can only be postulated that the lower doses delivered are actually the reason for this. Larger cavity size has been found to be associated with higher local failure rates in some retrospective analyses [29, 32, 34]. The rather broad dose corridors allowed within the study represent a potential limitation of the study, yet this issue will be addressed in a predefined subgroup analysis, as well as the cavity size. Addressing further limitations of the study, a possible confounding variable might also be the variation in margins. A resection cavity to CTV margin is constantly applied, yet for HFSRT the expansion might not necessarily be uniform for every patient, depending on the site of the resection cavity. In accordance with clinical practice, smaller safety margins are applied for SRS than for HFSRT. This factor might also be responsible for the relatively higher local failure rate in the single-dose trials [5, 6].

Designed to prospectively demonstrate the superiority of HFSRT over SFRT regarding local tumor control, the SATURNUS study applies a uniform target volume definition but allows for slight variations within dose prescription and CTV definition where needed to mirror a ‘real world setting’.

To the best of our knowledge, the SATURNUS trial is the only randomized phase III study comparing different techniques of postoperative stereotactic radiotherapy after resection of brain metastases adequately powered to detect a superiority of HFSRT regarding LC.

## Data Availability

Not applicable.

## Abbreviations

AE: Adverse Event
CI: Confidence Interval
CR: Complete response
CTV: Clinical target volume
DSGVO: Datenschutz-Grundverordnung (General Data Protection Regulation)
FU: Follow up
HFSRT: Hypofractionated stereotactic radiotherapy
IDL: Isodose line
IRB: Institutional Review Board
KPS: Karnofsky Performance Score
LC: Local Control
LD: Longest diameter
LRC: Locoregional Control = central nervous system progression free survival
OS: Overall Survival
PD: Progressive Disease
PI: Principal Investigator
PR: Partial response
PTV: Planning target volume
RAF: Response Assessment Form
RC: Resection cavity
RCV: Resection Cavity Volume
SAE: Severe Adverse Event
SRS: Single fraction stereotactic radiosurgery
SC: Study Coordinator
SD: Stable disease
TUM: Technical University of Munich
WBRT: Whole brain radiotherapy
